# 2588. Burden of Mortality and Antimicrobial Resistance of Bacterial Pathogens Associated with Lower Respiratory Tract and Other Thorax Infection in United States in 2019: A Systematic Analysis

**DOI:** 10.1093/ofid/ofad500.2203

**Published:** 2023-11-27

**Authors:** Sahithi Rao Mallyala, Avinash Chirumamilla, Deepti Raj, Jay Gajjar, Sneh Patel, Anushka Dekhne, Hardik Dineshbhai Desai

**Affiliations:** Dr. NTR University of Health Sciences, Vijaywada, Andhra Pradesh, India; Yenepoya Medical College, Manglore, Karnataka, India; K.S. Hegde Medical Academy, Manglore, Karnataka, India; Smt. NHL Municipal Medical College, Ahmedabad, Gujarat, India; GMERS Medical College, Gandhinagar, Gandhinagar, Gujarat, India; American University of Antigua, St Johns, Antigua, Osbourn, Saint John, Antigua and Barbuda; Gujarat Adani Institute of Medical Sciences, Affiliated K.S.K.V University, Ahmedabad, Gujarat, India

## Abstract

**Background:**

Detailed information about the impact of bacterial antimicrobial resistance (AMR) on Lower Respiratory Tract and other thorax Infections (LRTIs) is currently unavailable. It is important to have precise data on bacterial AMR to develop effective programs and policies for controlling AMR and to use antibiotics prudently for optimal treatment of LRTI patients. The primary objective of this research is to present extensive estimates of the mortality due to "bacterial pathogens" and its "AMR" of LRTIs in the United States in 2019.

**Methods:**

Mortality data associated with bacterial antimicrobial resistance and lower respiratory tract infection (LRTI) pathogens were acquired from the Global Burden of Diseases, Injuries, and Risk Factors Study (GBD) in 2019.

**Results:**

In United States, there were 88089 deaths (95% uncertainty interval [UI]: 66909-117145) due to bacterial pathogens in LRIT in 2019. Highest number of deaths observed due to Staphylococcus aureus (34.04%), followed by Streptococcus pneumoniae (18.30%), Pseudomonas aeruginosa (12.48%) in LRTI in 2019. There were 10984 deaths attributed to and 46482 deaths associated with bacterial AMR in LRTI in 2019.

**Figure:**
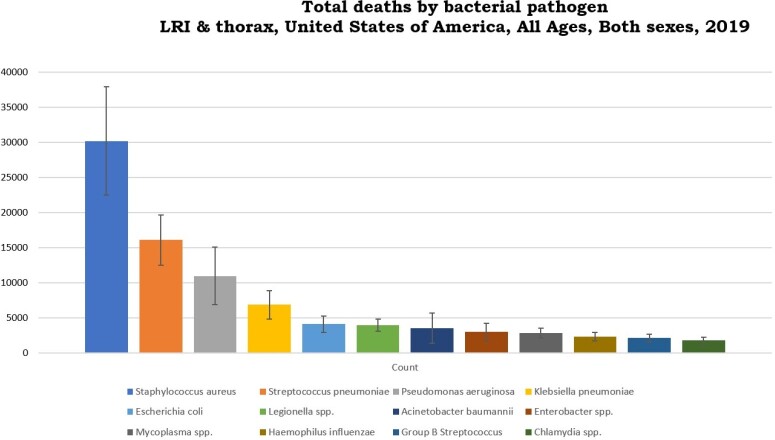

**Figure:**
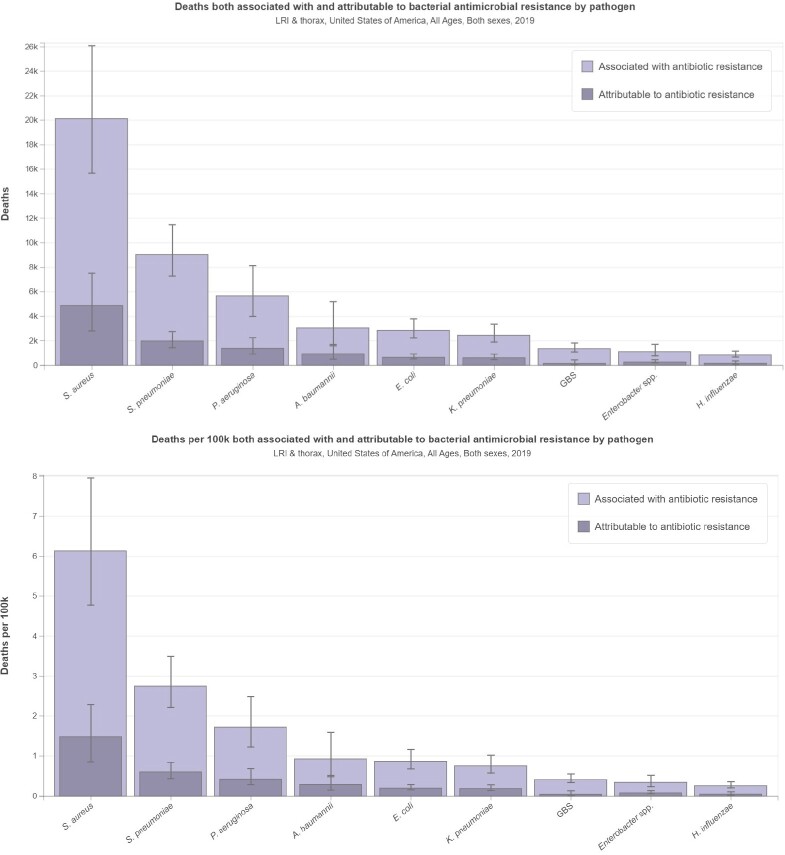

**Figure:**
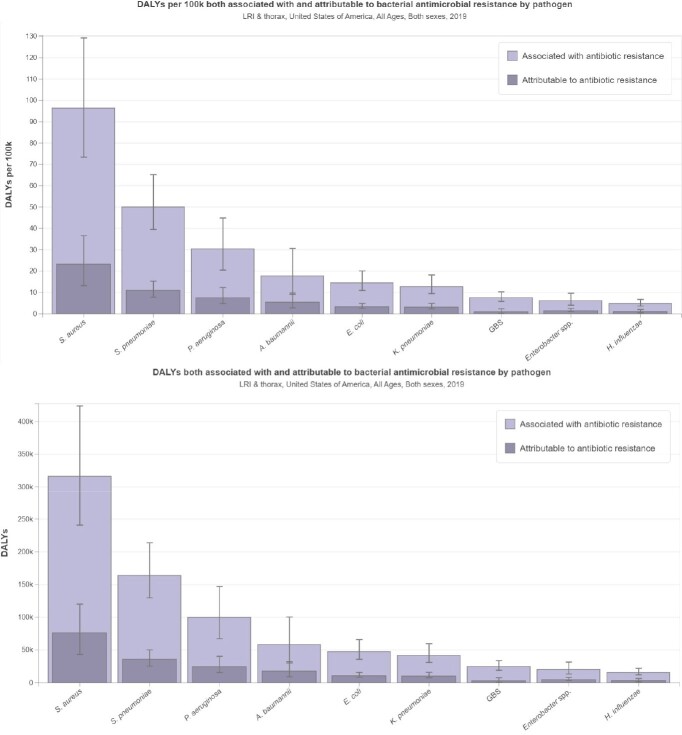

**Conclusion:**

Bacterial pathogens causing LRTIs are a significant cause of mortality in the United States. Moreover, antimicrobial resistance (AMR) among these pathogens has become a growing concern, exacerbating the burden of LRTI-related deaths. The implementation of such measures, along with the promotion of antimicrobial stewardship, can help to mitigate the impact of LRTI-associated deaths and preserve the effectiveness of available antimicrobial agents for the future.

**Disclosures:**

**All Authors**: No reported disclosures

